# Adverse Pregnancy Outcomes and *Coxiella burnetii* Antibodies in Pregnant Women, Denmark

**DOI:** 10.3201/eid2006.130584

**Published:** 2014-06

**Authors:** Stine Yde Nielsen, Kåre Mølbak, Tine Brink Henriksen, Karen Angeliki Krogfelt, Carsten Schade Larsen, Steen Villumsen

**Affiliations:** Aarhus University Hospital, Aarhus, Denmark (S.Y. Nielsen, T.B. Henriksen, C.S. Larsen);; Statens Serum Institut, Copenhagen, Denmark (K. Mølbak, K.A. Krogfelt, S. Villumsen)

**Keywords:** Q fever, Coxiella burnetii, Denmark, pregnancy, fetus, infant, mother, bacteria, zoonoses, infertility, seropositivity, antibodies, miscarriage

## Abstract

A high risk for obstetric complications has been reported among women infected with *Coxiella burnetii*, the causative agent of Q fever, but recent studies have failed to confirm these findings. We reviewed national data collected in Denmark during 2007–2011 and found 19 pregnancies in 12 women during which the mother had a positive or equivocal test for antibodies to *C. burnetii* (IgM phase I and II titers >64, IgG phase I and II titers >128). Of these 12 women, 4 experienced obstetric complications (miscarriage, preterm delivery, infant small for gestational age, oligohydramnion, fetal growth restriction, or perinatal death); these complications occurred in 9 pregnancies (47% of the 19 total pregnancies identified). Our findings suggest an association between Q fever and adverse pregnancy outcomes, but complications were identified in only 9 pregnancies during the study’s 5-year period, indicating that the overall risk is low.

## Abstract 2

Q fever may be associated with complications, but overall risk is low.

Q fever is a zoonotic infection caused by *Coxiella burnetii*. Findings of adverse pregnancy outcome in infected women, high seroprevalence in animal studies, and large human outbreaks have placed increasing focus on Q fever in several European countries, including Denmark (*1*–*4*). In ruminants, infection with *C. burnetii* is associated with high numbers of bacteria in the placenta, and the infection is known to cause abortion, retained placenta, endometritis, and infertility (*5*,*6*). Humans are infected with *C. burnetii* predominantly by inhalation of contaminated aerosols, and persons who have contact with livestock are at highest risk for exposure (*7*). Among pregnant women, >90% of those who show antibodies for *C. burnetii* that suggest recent infection may remain asymptomatic (*8*). Case series from France have associated symptomatic and asymptomatic *C. burnetii* infection during pregnancy with obstetric complications, including miscarriage, preterm delivery, and fetal death (*9*–*11*). In contrast, population studies from northern Europe have not found an association between *C. burnetii* and adverse pregnancy outcomes (*12*–*15*). 

Cattle are the main reservoir for *C. burnetii* in Denmark. A recent study of the seroprevalence of *C. burnetii* in cattle found that bulk-tank milk samples tested positive for *C. burnetii* at 59 of 100 randomly selected farms (*16*). In addition, the reported prevalence of antibodies to *C. burnetii* among veterinarians in Denmark ranges from 36% to 47% (*1*,*2*,*17*). These findings show that exposure to *C. burnetii* is common in this country in the animal reservoir and in those who are occupationally exposed to livestock or who live in rural areas with livestock contact. However, the risk for and implications of infection with *C. burnetii* among pregnant women have not been exhaustively described (*12*,*15*). Because of this, and because findings from the case series in France conflict with results from population-based studies from the Netherlands and Denmark, we reviewed national data from Aarhus University Hospital, Aalborg University Hospital, Hospital of Southwest Jutland, Viborg Regional Hospital, Regional Hospital West Jutland, and Hilleroed Hospital in Denmark to identify women who had elevated antibodies to *C. burnetii* during pregnancy. We evaluated the course of infection, effects of treatment with cotrimoxazole (trimethoprim-sulfamethoxazole), and pregnancy outcomes for these women. 

## Materials and Methods

Every resident in Denmark is provided with a unique civil registration number that enables individual-level linkage between national registries. Data from health records at obstetric and infectious disease departments were thereby linked to civil registration numbers from women (18–45 years of age) who had positive or equivocal tests at the Statens Serum Institut for antibodies to *C. burnetii* during 2007–2011. Using these data, we identified pregnant women who could be included in the study on the basis of positive serologic test results for *C. burnetii* and availability of titers from throughout pregnancy to enable evaluation of infection in paired samples.

### Detection of Antibodies against *C. burnetii*

In Denmark, *C. burnetii* serologic testing is performed only at the Statens Serum Institut by indirect immunofluorescence assay (IFA; Focus Diagnostics, Cypress, CA, USA), according to the manufacturer’s instructions. *C. burnetii* expresses 2 antigens, phase I and phase II. During active infection, phase II IgG and IgM are elevated; these results may remain positive for months to years. In acute Q fever, primarily antibodies against phase II antigens are raised, and these titers are higher than for antibodies against phase I antigens; IgM antibodies appear first. In chronic forms of the disease, antibodies against phase I antigens are elevated.

A local cutoff value adjusted to the population of Denmark has defined negative, equivocal, and positive titers (*18*); we included patients with equivocal and positive titers in our study. A sample was considered IFA-positive when IgM for phase I or phase II titers was >64 or IgG for any of the phases was >128. A 4-fold increase in titers between 2 paired samples was defined as diagnostic for recent or acute *C. burnetii* infection.

### PCR Analysis

DNA from urine samples was subjected to a Chelex 100-based DNA extraction method as described (*19*). DNA from placenta and bone marrow samples was extracted by using the DNeasy Blood and Tissue Kit (QIAGEN, Hilden, Germany) according to the manufacturer’s instructions. DNA from the cream layer of fresh breast milk samples was extracted by using a previously described protocol that included washing with phosphate-buffered saline and subsequent extraction with the DNeasy Blood and Tissue Kit (*20*). PCR was conducted with primers targeting the multicopy gene IS*1111* as described (*21*).

### Testing Indications and Pregnancy Outcomes

The indication for Q fever testing for most of the women was exposure to livestock. Two of the women were tested in a subsequent pregnancy because of a previous adverse pregnancy outcome; none were tested because of symptoms. Adverse pregnancy outcomes were defined as miscarriage, preterm delivery, single fetal death with a surviving co-twin, infant small for gestational age, oligohydramnion, fetal growth restriction, and perinatal death.

## Results

We identified 12 women with equivocal and positive antibody titers for *C. burnetii* infection who underwent 19 pregnancies during the 5-year study period. All women were farmers or veterinarians and resided in rural areas of Denmark. Obstetric complications were recorded in 9 (47%) of the 19 pregnancies (Table 1). None of the women were found to be IgM positive for pathogens regarded as classic causes of infection of the developing fetus during pregnancy; these pathogens included *Toxoplasma gondii*, parvovirus B19, rubella virus, cytomegalovirus, and herpes simplex virus. However, not all women were exhaustively tested during pregnancy. For all women tested during pregnancy, *C. burnetii* serologic test results were available from no later than pregnancy week 13 (Table 2).

**Table 1:** Patient characteristics and pregnancy outcomes in 12 women who had positive *Coxiella burnetii* titers during pregnancy, Denmark*

Pt and preg no.	Patient data					Fetal gestational age at delivery, wk + d	Baby birth weight, g	Clinical outcome of pregnancy	*C. burnetii* test results
	Age, y	Parity	Symptoms	Animal contact	Treatment during preg				
Pt 1	33	2	Fever and cough first weeks of pregnancy	Yes, cattle, vet	Yes, from wk 15	38 + 6	3,570	Healthy baby	PCR urine wk 10 pos; bone marrow biopsy wk 15 neg; PCR breast milk, amniotic fluid, placenta neg
Pt 2†	40	1	Dry cough for weeks just before first pregnancy	Yes, cattle, vet	No	8 wk	NA	Miscarriage	NA
Pt 3	30	1	No	Yes, cattle, vet	Yes, from wk 10	39 + 1	3,500	Healthy baby	PCR placenta neg
Pt 4‡	34	1	No	Yes, cattle, vet	No	39 + 0	3,030	Single fetal death around wk 8; surviving twin with healthy outcome	PCR breast milk, placenta neg
Pt 5§									
Preg 1	32	1	No	Yes, cattle, vet	No	40 + 3	3,000	Dysmature baby	NA
Preg 2	33	2	No		No	38 + 0	2,360	Caesarean, IUGR (23% from wk 28) and oligo	
Pt 6	26	1	NA	Yes, cattle, farmer	No	39	3,720	Healthy baby	PCR placenta neg
Pt 7¶	32	1	No	Yes, cattle, vet	Yes, from wk 10	41	3,210	Acute caesarean due to uterine rupture	PCR placenta neg
Pt 8									
Preg 1	24	0	No	Yes, cattle, assisting female farmer	No	27 + 2	NA	IUGR, oligo/ malformations; baby died few hours postpartum	NA
Preg 2	26	1	No		Yes, from wk 22	30	1,570	Preterm baby	PCR placenta neg
Pt 9									
Preg 1	30	0	No	Yes, cattle, vet	From wk 20	39 + 4	3,790	Healthy baby	PCR placenta, breast milk neg
Preg 2	33	1	No		No	40 + 2	4,170	Healthy baby	NA
Pt 10									
Preg 1	30	0	1 mo dry cough at start of preg, short episode of fever	Yes, cattle, vet	Yes, from wk 10	39 + 6	3,420	Healthy baby	PCR placenta, breast milk neg
Preg 2	33	1	No		No	41 + 2	3,400	Healthy baby	NA
Pt 11	30	1	No	Yes, cattle, vet	No	40	4,230	Healthy baby	PCR placenta neg
Pt 12	31	1	No	Yes, cattle, vet	Yes, from wk 22	39 + 5	3,570	Healthy baby	PCR placenta neg

*See Table 2 for specific titers. Pt, patient; preg, pregnancy; vet, veterinarian; NA, not applicable; IUGR, intrauterine growth restriction; oligo, oligohydramnion. †Within 2 years, 3 spontaneous abortions and 1 extrauterine preg. ‡Q fever in 2006 (not pregnant). §Acute Q fever in 2006 (not pregnant), treated with 3 wk doxycycline. ¶Two spontaneous abortions before this preg.

**Table 2:** Results of testing for 12 women who had positive *Coxiella burnetii* titers during pregnancy, Denmark*

Pt and preg	First sample							Last sample					Comments
	Date collected†	Titers						Date collected†	Titers				
		IgM II	IgM I		IgG II	IgG I			IgM II	IgM I	IgG II	IgG I	
Pt 1	7 wk	2,048	8,000		1,024	512		38 wk	<64	<64	256	<128	Negative titers 6 mo before this preg
Pt 2	8 wk, after miscarriage	256	<64		256	128		7 wk, after extrauterine preg	512	128	512	512	Maximum titers after second miscarriage: IgG phase II, 4,096; IgG phase I, 2,048; IgM phase I, 512; IgM phase II, 256
Pt 3	3 wk	<64	<64		<128	512		33	<64	<64	128	1,024	First titers taken 2 mo before this preg: IgG phase I, 1,024. Maximum titers in preg week 9: IgG phase I, 4,096; IgG phase II, 256‡
Pt 4	10 wk	<64	<64		1,024	512		1 day postpartum	<64	<64	1,024	128	First titers taken 3 mo before this preg identical to titers from preg week 10§
Pt 5													
Preg 1	9 wk	<64	<64		256	512		38 wk	<64	<64	256	256	First titers taken 6 mo before this preg: IgM phase II, <64; IgM phase I, <64; IgG phase II, 512; IgG phase I, 1,024§
Preg 2	12 wk	<64	<64		128	1,024		31 wk	<64	<64	512	1,024	§
Pt 6	13 wk	<64	<64		256	<128		At birth	<64	<64	128	<128	No titers available before this preg. Maximum titers in preg: IgG phase II, 256‡
Pt 7	8 wk	128	<64	4,096		<128		At birth	64	<64	512	<128	Titers positive 6 mo before this preg: IgM phase II, 128; IgM phase I, <64; IgG phase II, 2048; IgG phase I, <128§
Pt 8													
Preg 1	12 wk	128	<64	512		256		26 wk	256	<64	1,024	256	Maximum titers in this preg: IgG phase II, 2,048‡
Preg 2	10 wk	<64	<64	256		128		26 wk	<64	<64	256	256	Maximum titers in this preg: IgG phase I, 1,024; IgG phase II, 1,024‡
Pt 9													
Preg 1	10 wk	<64	<64	256		<128		39 wk	64	<64	128	<128	Maximum titers in this preg: phase II IgG, 1,024‡
Preg 2	12 wk	<64	<64	256		<128		36 wk	<64	<64	256	<128	§
Pt 10													
Preg 1	8 wk	512	<64	4,096		<128		36 wk	<64	<164	256	<128	No titers available before this preg§
Preg 2	9 wk	<64	<64	128		<128		14 wk	<64	<64	<128	<128	§
Pt 11	7 wk	<64	<64	128		128		26 wk	256	256	512	<128	Titers positive 1 mo before this preg: IgM phase II, <64; IgM phase I, <64; IgG phase II, 1,024; IgG phase I, <128§
Pt 12	10 wk	<64	<64	2,048		<128		37 wk	<64	<64	1,024	1,024	Negative titers during preg 2 years earlier. Maximum titers in this preg: IgG phase II, 2,048

*See Table 1 for specific patient and outcome data. Pt, patient; preg, pregnancy; vet, veterinarian; NA, not applicable; IUGR, intrauterine growth restriction. †Gestational week. ‡Remaining titers have not risen above/beyond values in beginning/end of pregnancy. §No further rise of titers in this pregnancy; indicates that titers had not risen beyond values at beginning/end of pregnancy.

Two patients (1 and 10) reported dry cough and short episodes of fever; both had antibody titers during the first trimester consistent with acute infection. These patients were treated with cotrimoxazole beginning in gestational week 15 or 10, respectively; patient 1 was treated throughout pregnancy and patient 10 until gestational week 39. Patent 1 had a PCR-positive urine sample in gestational week 10, but results of PCR on a bone marrow biopsy from gestational week 15 and PCR on amniotic fluid and placenta were negative. For patient 10, no *C. burnetii* DNA was detected by PCR from placental tissue or breast milk; for her second pregnancy, serologic test results for *C. burnetii* were negative, PCR on placenta was not performed, and the pregnancy had a healthy outcome.

Patient 2 reported weeks of dry cough without fever during the weeks just before the first pregnancy that ended with miscarriage. She had 3 miscarriages and an extrauterine pregnancy within 2 years, and the titers that were found indicate that she was acutely infected weeks before the first miscarriage. Her antibody titers reached a maximum level after the second miscarriage. No embryo material was tested from any of her miscarriages.

Patients 3, 5, and 12 had serologic profiles with IgG phase I titer of 1,024 at the end of pregnancy; 2 of the patients were treated during pregnancy, but none had symptoms, received a diagnoses of endocarditis, or received long-term postpartum treatment. Patient 12 had a serologic profile indicating reactivation of *C. burnetii* infection, but her antibody titers had been negative in a previous pregnancy 2 years earlier.

Patient 4 seroconverted before her second pregnancy, during which she experienced a single fetal death around gestational week 8 and a surviving co-twin. She had a decrease in IgG phase I during pregnancy; the surviving twin was delivered healthy and at term.

Patient 5 was treated for acute Q fever before her second pregnancy. Her antibody titers were stable during the second pregnancy, and her baby was full-term but slightly small for gestational age. After a short interpregnancy interval, she had a significant increase in IgG phase II titers during her third pregnancy; because of fetal growth restriction and oligohydramnion noted during gestational week 28, she had a cesarean section during gestational week 38. The placentas from these 2 pregnancies were not tested.

Patient 8 had rising antibody titers during her first pregnancy, and because of bleeding and contractions, she had an acute cesarean section in week 27 and gave birth to a severely growth retarded and malformed infant who lived only a few hours. The fetus and placenta were not tested for *C. burnetii*, but results of testing for toxoplasmosis, cytomegalovirus, and parvovirus B19 were negative, as were results of genetic testing for neuromuscular diseases. Her titers decreased slightly postpartum, but during her second pregnancy, titers increased significantly, and treatment with cotrimoxazole was initiated around gestational week 22. In gestational week 30, she spontaneously went into labor and gave birth to a healthy baby. Treatment was terminated immediately postpartum, and her antibody levels decreased, indicating that she was not chronically infected. Thus, patients 5 and 8 had serologic indication of a reactivation of infection with *C. burnetii*: a postpartum decline in antibody titers followed by a >4-fold increase in titers during the next pregnancy.

Three of the remaining pregnancies (in patients 6, 9, and 11) had an uncomplicated course with a healthy pregnancy outcome. Patient 7 had an acute cesarean because of rupture of the uterus; she had had a cesarean in her first pregnancy.

In summary, 3 patients (1, 2, and 10) reported symptoms of acute Q fever. At least 1 (patient 8) appeared to have seroconverted without symptoms close to the beginning of her first pregnancy. 

For 7 of the 19 pregnancies, treatment with cotrimoxazole was initiated in the patient; 6 of these pregnancies resulted in healthy, full-term babies, and no mention of severe side effects was found in the mothers’ medical records. One of the women who had obstetric complications received treatment with cotrimoxazole (patient 8, in her second pregnancy). By comparison, among the 12 pregnancies in which no treatment was given, 8 resulted in obstetric complications. The effect of treatment with cotrimoxazole on complications was tested, but the difference was not significant (p = 0.057 by Fisher exact test; data not shown).

PCR was performed on placentas from 10 pregnancies and in 4 of these, breast milk was also tested; no results were positive. For 7 of the 10 pregnancies in which placentas were tested, the woman had received treatment with cotrimoxazole during pregnancy.

## Discussion

Adverse pregnancy outcome was observed in 9 of 19 pregnancies among 4 of the 12 pregnant women in which equivocal or positive tests for *C. burnetii* antibodies were found. One woman had 3 miscarriages and an extrauterine pregnancy, 1 experienced preterm delivery, 1 had a single fetal death with a surviving co-twin, and 1 delivered a small-for-gestational-age baby. Oligohydramnion and fetal growth restriction were found in 2 pregnancies; 1 had a healthy outcome, but in the other, the baby died a few hours postpartum.

The observed complication rate of 47% may seem high, but the causal relationship of this finding may not be clear. For example, none of the tested placentas were examined by histopathology, and all of the 10 placentas tested by PCR were negative for *C. burnetii*. Furthermore, not all of the women were thoroughly tested for other infections. One possible explanation for the lack of findings related to the placenta in 7 of the cases could be the patient’s treatment with cotrimoxazole or focal placental infection. Nonetheless, we did observe adverse pregnancy outcome in 8 (67%) of 12 pregnancies in which the women were not treated with cotrimoxazole, which supports the beneficial effects of treatment.

Among the cases we reviewed, none of the 4 breast milk samples tested by PCR were positive for *C. burnetii*. This bacterium has been found in human milk (*22*,*23*), but the implications for the breastfed child are unclear. Because of the lack of evidence, breastfeeding has been deemed safe according to the obstetric guidelines in Denmark for the treatment of *C. burnetii*–seropositive pregnant women and their newborns.

Two studies, in the Netherlands (*24*) and Canada (*25*), have suggested a low rate of placental *C. burnetii* infection in asymptomatic women and that obstetric complications in symptomatic cases may be explained by massive placental necrosis following a higher bacterial load in the placenta, systemic infection, or both. A study in France (*26*) found that untreated Q fever in 1 pregnancy may be reactivated in a subsequent pregnancy, a result found in 3 patients in our study (patients 5, 8, and 12).

The evidence for an adverse pregnancy outcome in humans in relation to Q fever mainly originates from case studies from France of referred, infected, pregnant patients, as well as pregnancies in which a diagnosis of Q fever was reached retrospectively, after an adverse outcome (*9*–*11*). Carcopino et al. reported clinical symptoms in 32 (60.4%) of 53 cases and a chronic serologic profile in more than half of patients and concluded that Q fever in pregnancy may cause severe complications (*10*). The Netherlands has recently experienced an unprecedented Q fever outbreak that has prompted 2 large studies in pregnant women. One study, a population-based study of 1,174 serum samples collected at the twelfth week of pregnancy, found no association between antibodies to *C. burnetii* and adverse pregnancy outcome among women living in the area with the highest Q fever incidence. The other study, a randomized, controlled trial, tested 1,229 pregnant women living in high-risk areas during the outbreak; 15% of the women were seropositive in both the intervention group and the control group, and no difference was found in obstetric complications (*13*). Hence, the findings from France were not reproduced in the Netherlands. Likewise, a recent study in Denmark assessed the association between presence of antibodies to *C. burnetii*, seroconversion, and pregnancy outcome and found that seropositivity was not associated with miscarriage, preterm birth, or low birthweight (*15*).

Differences in findings among these various studies may be explained in part by differences in study design. The indication for serologic testing is a crucial point; in our study, the indication for testing was exposure to livestock for most of the women. In contrast, the women in the case series in France were primarily tested because of pathologic conditions during pregnancy or clinical symptoms (e.g., fever, hepatitis) or retrospectively because of an adverse pregnancy outcome. Angelakis et al. (*11*) found that 17 of 30 *C. burnetii* seropositive pregnant women were asymptomatic; only 2 of these had an uncomplicated pregnancy, but no placentitis or isolation of *C. burnetii* was found in 14 available biopsy specimens. These authors suggested that the different rates of obstetric complications found in various geographic areas could be related to strain specificity, potentially because of differences in plasmid types.

All the women in this case series had contact with livestock in Denmark, and it is reasonable to assume that these women were occupationally exposed to endemically occurring *C. burnetii* infections among cattle. However, the observations by Angelakis et al. suggest that strains of *C. burnetti* in Denmark, and possibly cattle in general, might be less virulent than that seen when the infection is acquired from other animal reservoirs (e.g., goats). In France, goats and sheep have been the main source of *C. burnetti* infection. The recent outbreak in the Netherlands was detected shortly after a large number of dairy milk farms had changed from cattle to goats as production animals. In Denmark, goat and sheep farms are rare, and despite high clinical awareness during the past 7 years, no reports have described a microbiologically verified outbreak of Q fever in humans or a case of chronic Q fever that was definitely acquired in Denmark. However, a large percentage of dairy cattle in Denmark shed *C. burnetii*, and a high prevalence of antibodies has been found among pregnant women who had exposure to cattle (*16*,*17*). These observations could, in part, be an explanation for the discrepancies in rates of serious adverse pregnancy outcomes among studies from different countries.

In conclusion, we evaluated risks and implications in 19 pregnancies with positive or rising titers against *C. burnetii* from Denmark, a country that has high seroprevalence of *C. burnetii* but low prevalence of clinical Q fever and for which cattle are the primary bacterial reservoir. In this case series, almost half of the women had obstetric complications, which is comparable to previous case series. We found complications in 8 out of 12 untreated pregnancies; 7 pregnant women received long-term treatment with cotrimoxazole. In this study, serologic signs of Q fever were associated with adverse pregnancy outcome. However, in none of the cases could we identify a definite causal relationship between *C. burnetii* seropositivity and adverse pregnancy outcome. Because only 9 cases of adverse pregnancy outcome were found over 5 years, despite increased awareness among the relevant risk groups, and because community studies in Denmark and the Netherlands have failed to confirm this association, the overall risk for a Q fever–associated adverse pregnancy outcome in Denmark is likely to be low.
